# Exploring right ovary degeneration in duck and goose embryos by histology and transcriptome dynamics analysis

**DOI:** 10.1186/s12864-023-09493-0

**Published:** 2023-07-10

**Authors:** Mingxia Ran, Qingyuan Ouyang, Xuejian Li, Shenqiang Hu, Bo Hu, Jiwei Hu, Dan Dong, Liang Li, Hua He, Hehe Liu, Jiwen Wang

**Affiliations:** 1 Farm Animal Genetic Resources Exploration and Innovation Key Laboratory of Sichuan Province, Sichuan Agricultural University, Chengdu, 611130 China; 2 Yucheng District Agriculture and Rural Affairs Bureau, Ya’an, 625000 China

**Keywords:** Right ovary degeneration, Goose, Duck, Transcriptome sequencing

## Abstract

**Background:**

The development of asymmetric chick gonads involves separate developmental programs in the left and right gonads. In contrast to the left ovary developing into a fully functional reproductive organ, the right ovary undergoes gradual degeneration. However, the molecular mechanisms underlying the the degeneration of the right ovary remain incompletely understood. In the present study, we investigated the histomorphological and transcriptomic changes in the right ovary of ducks and geese during the the embryonic stage up to post-hatching day 1.

**Result:**

Hematoxylin–eosin stainings revealed that the right ovary developed until embryonic day 20 in ducks (DE20) or embryonic day 22 in geese (GE22), after which it started to regress. Further RNA-seq analyses revealed that both the differentially expressed genes (DEGs) in ducks and geese right ovary developmental stage were significantly enriched in cell adhesion-related pathway (ECM-receptor interaction, Focal adhesion pathway) and Cellular senescence pathway. Then during the degeneration stage, the DEGs were primarily enriched in pathways associated with inflammation, including Herpes simplex virus 1 infection, Influenza A, and Toll-like receptor signaling pathway. Moreover, duck-specific DEGs showed enrichment in Steroid hormone biosynthesis, Base excision repair, and the Wnt signaling pathway, while geese-specifically DEGs were found to be enriched in apoptosis and inflammation-related pathways, such as Ferroptosis, Necroptosis, RIG-I-like receptor signaling pathway, and NOD-like receptor signaling pathway. These findings suggest that the degeneration process of the right ovary in ducks occurs at a slower pace compared to that in geese. Additionally, the observation of the left ovary of the geese varying degeneration rates in the right ovary after hatching indicated that the development of the left ovary may be influenced by the degeneration of the right ovary.

**Conclusion:**

The data presented in this study provide valuable insights into the dynamic changes in histological structure and transcriptome during the degeneration of the right ovary in ducks and geese. In addition, through the analysis of shared characteristics in the degeneration process of the right ovary in both ducks and geese, we have uncovered the patterns of degradation and elucidated the molecular mechanisms involved in the regression of the right ovary in poultry. Furthermore, we have also made initial discoveries regarding the relationship between the degeneration of the right ovary and the development of the left ovary.

**Supplementary Information:**

The online version contains supplementary material available at 10.1186/s12864-023-09493-0.

## Background

Vertebrate bodies are organized along three orthogonal axes: anterior–posterior, dorsal–ventral, and left–right (L-R). Under the complex genetic interactions, during the intricate process of L-R axis development, the organs in vertebrates intricate process of L-R asymmetry [1]. Except in birds of some prey, avian gonads typically develop asymmetrical ovaries, with only one ovary undergoing successful development [2, 3]. Therefore, the morphological development of the left and right gonads in birds has been considered a model system to underlying the molecular basis of the asymmetry development. In chickens, the development of asymmetric ovaries can be observed as early as embryonic day 7 (E7). During this stage, the left gonad undergoes specific changes. Cortical cords extend inward from the germinal epithelium, giving rise to the formation of the cortex and medullary lacunae give rise to the medulla, somatic and germ cells proliferate within the cortex [4]. While the right gonad also forms a vacuolated medulla, it fails to form a thickened germ cell-enriched cortex at E7 since it does not form a cortical cord. This is the main reason why the right gonadal fails to develop into a fully functional ovary [3, 5–7]. According to Gonzalez-Morán MG’s reportes, the right ovary in chickens lacks a cortical layer. However, the components of the right ovarian medulla advanced development until the age of 1-day old and then regressed. As a result, there is a reduction in the the total volume of blood vessels, lacunar channels. During the first four weeks post-hatching, the right ovary of the chickens was dominated by interstitial cells [8]. However, Yu Ping observed the slices of the right ovary of the 7.5 to 13.5 embryonic age of the Sea Orchid chicken and found that the right ovary started to exhibit lacunar channels structures from E7.5, and the number and volume of these structures gradually increased with the age of the embryo. By E13.5, these structures nearly occupied the entire right ovary, but there was no significant change in volume, indicating that the right ovary began to degenerate after gender differentiation and did not have a developmental stage [9]. This observation suggests that the right ovary may began to degeneration from the sex differentiation, then reaching near complete degradation prior to the emergence from the eggshell, which is differs from the findings of Gonzalez-Morán MG et al. Therefore, there is still controversy over when the right ovary of poultry began to degenerate.

Throughout embryogenesis, the right female gonad expresses essential female marker genes although it does not develop into a functional ovary and ultimately regresses.This observation suggests that both the right and left gonads initially engaged in the ovary-determining pathway [10]. Furthermore, during the embryonic period, the right ovary of chickens has also been detected to secrete androgens, estrogens, and progesterone, and is responsiveto chorionic gonadotropin stimulation [11]. Moreover, when the right ovary is surgically removed, the right ovary of young chick undergoes compensatory growth and assumes the structure of an immature testis. Importantly the regression of the Müllerian duct continues normally even after the removal of the left ovary. These findings indicate that the right ovary secretes sufficient hormones to induce degeneration of the Müllerian duct [10, 12, 13]. Steroid hormones play a critical role in regualting ovarian development and egg production [10, 14], and these results suggest that the right ovary may have an impact on avian reproductive performance before degeneration like the left ovary. Therefore, it is imperative to continue exploring the degradation law of the right ovary and its molecular mechanism, so as to lay a foundation for analyzing its relationship with the development of the left ovary.

Most researchs investigating the molecular mechanisms underlying right ovarian degeneration have primarily focused on asymmetric development following sex differentiation. Following gastrulation, the pathway governing asymmetric operates through a cascade of asymmetric molecular signals that initiate at the node and propagated towards the gonad. One of the downstream target of this asymmetric signaling in the gonad is the transcription factor *PITX2 *[15, 16]. *PITX2*, a member of the *RIEG/PITX* homeobox family, exhibits exclusive expression in the left ovary. By inhibiting retinal dehydrogenase RALDH2 expression, *PITX2* reduces retinoic acid (RA) signaling and the expression of RA nuclear receptor Ad4BP/SF-1. Consequently, this results in an increase in estrogen receptor (ER) expression and promotes cortical cell proliferation in the left ovary. In contrast, the absence of *PITX2* expression in the cortical layer of the right ovary leads to reduced RALDH2 expression. This reduction, in turn, activates of RA signaling, inhibites the expression of Ad4BP/SF-1, ER and cyclin D1 expression, ultimately leading to the inhibition of cortical cell proliferation in the right ovary [1, 17, 18]. Moreover, the expression of *PITX2* on the right side before and during gonadogenesis is sufficient to transform the right gonad into a left-like gonad [19]. Therefore, *PITX2* may serve as the initiator of the mechanism that actively promotes underlying L-R asymmetry in females. Wang et al. performed RNA-Seq analysis on total RNA harvested from the left and right gonads at embryonic day 6 (E6), E12, and post-hatching day 1 (D1). This study revealed that differentially expressed genes between the left and right gonads were annotated to the piwi-interaction RNA (piRNA) metabolic pathway [20]. *PIWIL1* plays a central role in gametogenesis by repressing transposable elements and preventing their mobilization. This suggests that piRNAs may have significant roles in maintenaning avian ovarian asymmetry [3, 21]. However, the molecular mechanism governing right ovarian degeneration have yet to be thoroughly investigated.

In summary, further research is needed to explore the degradation patterns and molecular mechanisms underlying the degeneration of the right ovary in poultry species. In present research, we have chosen ducks (Anas platyrhynchos) and geese (Anser cygnoides) as our research subjects, to explore the dynamic changes of histological structure and molecular mechanisms during right ovary degeneration using hematoxylin–eosin (HE) staining and RNA-seq. It is expected to further investigate the degradation patterns and molecular mechanisms underlying the degeneration of the right ovary through analyzing the common of right ovary degeneration between different poultry. Moreover, by comparing the development of the left ovary in geese vary in degree of right ovary degeneration, the relationship between the right ovary degeneration and the development of the left ovary was preliminarily explored. The purpose of this study is to clarify the degradation law and molecular mechanism of the right ovary in poultry, and further lay the foundation for the study of the relationship between the right ovary degradation and the left ovary development.

## Methods

### Animals and sample collection

All the female Tianfu Meat geese and Nonghua Sheldrake ducks used in this study were hatched from the same batch of fertilized eggs obtained from the Waterfowl Breeding Experimental Farm of Sichuan Agricultural University (Ya’an campus, Sichuan, China). Sex identification was performed using PCR amplification of the chromodomain helicase DNA binding protein 1 (CHD1) gene sequence on embryonic brain/heart tissue. The primer set used for amplification was as follows: Forward primer: 5’-TGCAGAAGCAATATTACAAGT-3’; Reverse primer: 5’-AATTCATTATCATCTGGTGG-3’, as previously described in Liu et al. [22]. Thereafter, after measuring the weight, the left and right gonads were weighed and collected at specific time points: duck embryonic day 10 (DE10), DE20, and post-hatching day 1(DP1); goose embryonic stage 12 days (GE12), GE22, post-hatching day 1 (GP1), GP3, GP6. All selected geese post-hatch were euthanized by inhalation of carbon dioxide followed by cervical dislocation, performed by experienced personnel trained in the approptiate technique.

### Histomorphological observation

The left and right gonads for histological comparison (5 per group) were 4% formaldehyde-fixed for 72 h at room temperature. The fixed samples were then dehydrated and embedded using TSJ-II automatic closed tissue dehydrator (Changzhou Zhongwei Electronic Instrument Co., Ltd, Changzhou, China) and the BMJ-III embedding machine (Zhongwei electronic instrument factory in Changzhou suburb, Changzhou, China), respectively. A rotary microtome (Leica, Oskar-Barnack, Germany) was utilized to cut 4 μm thick sections from the specimens. The Cross-sections were further stained with hematoxylin and eosin (HE) and photographed using digital trinocular camera microscope BA410 Digital (Motic China Group Co. Ltd., Xiamen, China). Furthermore, the degeneration of the right ovary in the geese was observed based on anatomical and tissue sections. Geese with rapid or slow ovarian degeneration on the right side were selected (5 individuals per group). Geese showing no observed right ovary in the anatomy were classified as individuals with rapid degeneration, while geese with an evident right ovary in the anatomy and tightly organized histological structure were classified as individuals with slow degeneration. Then the long and short diameters of the left ovary, and the number of primordial follicles on GP6 were counted using Image-Pro Plus 6.0 (Media Cybernetics Company of America, MD, United States).

### Total RNA extraction, library preparation, and sequencing

The Trizol kit (Invitrogen, CA, United States) was used to extract the total RNA of ovaries according to the manufacturer’s instructions. The RNA purity was evaluated using the NanoPhotometerR spectrophotometer (IMPLEN, CA, United States). The QubitR RNA Assay Kit in QubitR 2.0 Fluorometer (Life Technologies, CA, United States) was used to measured the RNA concentration. The RNA integrity was assessed using the RNA Nano 6000 Assay Kit on the Bioanalyzer 2100 System (Agilent Technologies, CA, United States) [23].

A total amount of 20 ng RNA per sample was used as input material for RNA sample preparations. Firstly, Epicenter RiboZeroTM rRNA Removal Kit (Epicenter, WI, United States) was employed to remove ribosomal RNA, and the rRNA-free residue was cleaned up using ethanol precipitation. Subsequently, sequencing libraries were constructed using the rRNA-depleted RNA with the NEBNextR UltraTM Directional RNA Library Prep Kit for Illumina (New England Biolabs, Inc., MA, United States) following manufacturer’s recommendations. Briefly, fragmentation was carried out using divalent cations at elevated temperatures in NEBNext First Strand Synthesis Reaction Buffer (5X). The first-strand cDNA was synthesized using a random hexamer primer and M-MuLV Reverse Transcriptase (RNaseH-).

Then, following the second-strand cDNA synthesis performed by DNA polymerase I and RNase H, the dNTPs with dTTP were substituted with dUTP in the reaction buffer. Subsequently, the remaining overhangs were converted into blunt ends using exonuclease/polymerase activities. Finally, the 3’ ends of DNA fragments were adenylated, the NEBNext adaptor with a hairpin loop structure was ligated to prepare for hybridization. To selectively retain cDNA fragments of 150–200 bp, the library fragments were purified using the AMPure XP system (Beckman Coulter, Beverly, MA, United States). A total of 3 mL USER Enzyme (New England Biolabs, Inc., MA, United States) was used for size selection. Prior to the polymerase chain reaction (PCR) reaction, the adaptor-ligated cDNA was incubated at 37℃ for 15 min, followed by 5 min at 95℃. Then, Phusion High-Fidelity DNA polymerase, Universal PCR primers, and Index (X) Primer were added for PCR reaction. At last, the resulting PCR peoducts were putified using the AMPure XP system, and the library's quality was evaluated on the Agilent Bioanalyzer 2100 system. Before sequencing, the index-coded samples were clustered on a cBot Cluster Generation System using TruSeq PE Cluster Kit v3-cBot-HS (Illumina). After cluster generation, paired-end reads were generated by sequencing the libraries on an Illumina Hiseq 2500 platform [24]. All RNA-seq data obtained in the current study are available in the BioProject database under accessin number PRJNA821376.

### Quality analysis, mapping, and transcriptome assembly

The raw data in fastq format were initially processed through in-house Perl scripts. In this step, clean data (clean reads) were obtained by removing reads containing adapter, reads containing ploy-N, and low-quality reads. Reads shorter than 18nt or longer than 30nt were also trimmed and filtered out. The Bowtie software (version 1.2.3) was employed to align the clean reads according to Silva databases, GtRNAdb databases, Rfam databases, and Repbase databases. The genome of Zhedong white geese (Anser cygnoides) (GCF_000971095.1) and mallard (Anas platyrhynchos) (GCF_015476345.1) were used as the reference genome for both sequence alignment and subsequent analysis. The clean reads of ducks and geese were aligned to the Zhedong white geese (Anser cygnoides) genome and mallard (Anas platyrhynchos) genome, respectively, using the HISAT2 (v2.0.4) alignment tool [25]. The mapped reads of each sample were assembled by StringTie (v1.3.3) [24] using a reference-based approach.

### Differential expression analysis

The obtained read counts were normalized as transcripts per million (TPM). Differentially expression analysis of genes was performed using the DESeq2 package of R. The P values were adjusted using Benjamini and Hochberg's approach to control the false discovery rate. For biological replicates, mRNA expression differences were considered significant if the adjusted P-value (FDR) < 0.05 and log2|foldchange|> 1.5.

### Gene ontology and kyoto encyclopedia of genes and genomes enrichment analyses

Gene Ontology (GO) and Kyoto Encyclopedia of Genes and Genomes (KEGG) enrichment analyses were conducted by KOBAS 3.0 online tool [26]. The GO terms with acorrected *P*-Value < 0.05 and KEGG pathways with *P*-Value < 0.05 were considered significantly enriched by differentially expressed genes (DEGs).

### Quantitative real-time PCR validation

Nine DEGs were selected for PCR verification. The total RNA extracted from the right ovaries was reverse transcribed into cDNA using the PrimeScriptTM RT Reagent Kit (Takara Biotechnology Co., Ltd., Dalian, China). Furthermore, the quantitative PCR (qPCR) reactions were performed on the Bio-Rad CFX96 real-time PCR detection system (Bio-Rad, Hercules, CA, United States) using TB GreenTM Premix Ex Taq TM (Takara Biotechnology Co., Ltd., Dalian, China) [27] and the primer designed by Primer3Plus1 (Table 1). The relative expression levels were normalized to the reference genes *GAPDH* using the comparative Cq method (ΔΔCq) method. Statistical differences in genes expression were analyzed using a one-way analysis of variance (ANOVA) test in SPSS (version 20.0, IBM, IL, United States). All data were presented as the means ± SEM. *P*-value < 0.05 was considered statistically significant.

**Table 1:** Quantitative real-time PCR primers

Gene	**Forward Primer**	**Reverse Primer**	**Product size**	**Tm**
FN1	ACCAAGACACCAAGACTTCCTA	CATTCGCCTCTGCCATTACC	106	59
COL6A1	TGATTGTGGTGACGGATGGA	AGAGATGGCGACGGAGAAC	119	59
COL6A2	AGCCAGAACAGAGCGAGAC	AGCATGTCCTCCATCTTGTCA	121	59
STAT1	TTGCTACGGTGCTGTTCCA	TCCTGAAGGTTACGCTTGCT	121	52
B2M	CGACATCACCTTGCTGAAGAAT	GAAGGTCCAGTCGTCGTTGA	82	59
POSTN	CTTGAAGTTGGCTGTGATGGT	TTGGCAGAATCAGGAATTAGCA	128	58
MLF1	ACAGGAAGACTGGTGATGAAGA	ACGGCTTGAACTTGGTTATCTC	111	58
FHL2	GCAAGGATCTGTCTTACAAGGA	GTATTCGTTGGAGTAGCAGTCA	140	57
RNF213	GTCAACACAGGAGAGCACTTC	AGGCACAGGTATCTGAATGGTA	85	58
GAPDH	TTTCCCCACAGCCTTAGCA	GCCATCACAGCCACACAGA	90	60

## Results

### Histological changes of right ovary during its degeneration in ducks and geese

**Fig. 1:**
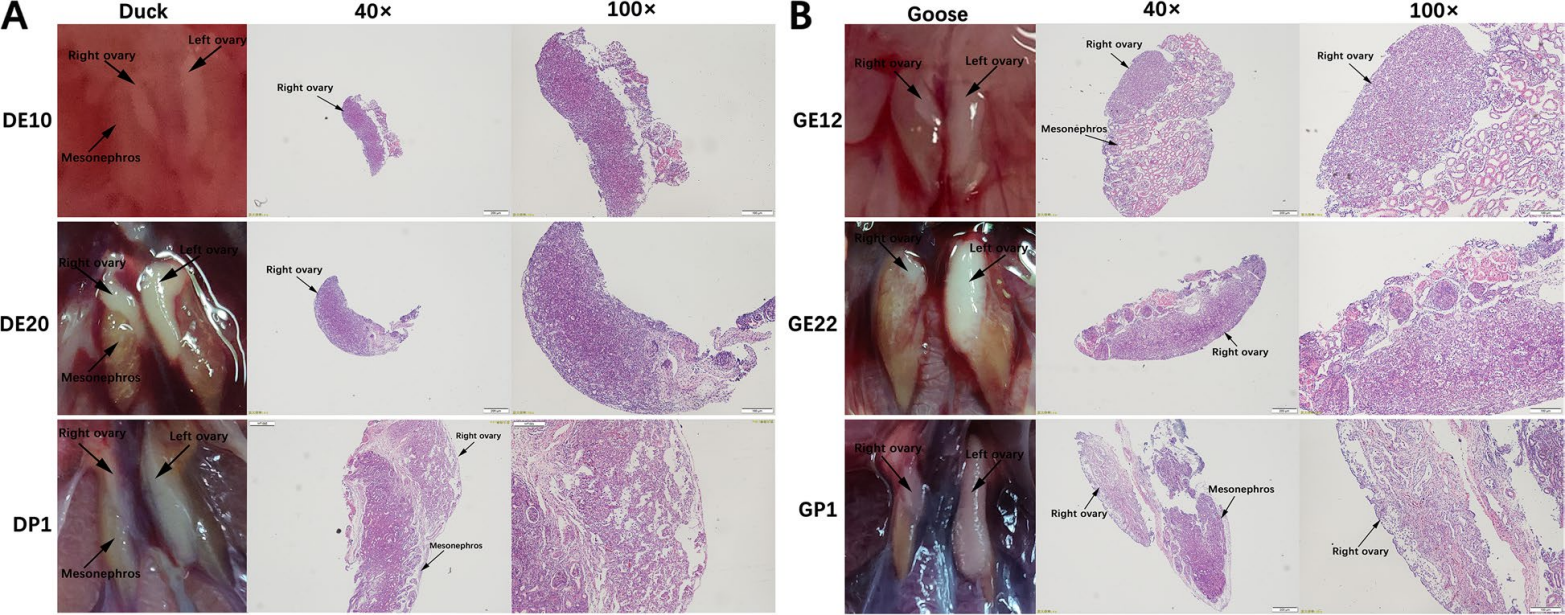
**A** and **B**: Anatomical pictures (Left) and photomicrograph of HE-stained right ovaries (Right) from the DE10, DE20, DP1, GE12, GE22, and GP1, respectively (DE: Duck embryonic, DP: Duck post-hatching, GE: Goose embryonic, GP: Goose post-hatching)

Anatomical and histological observations indicate that the ducks and geese exhibit a similar pattern of right ovarian degeneration: from DE10/GE12 to DE20/GE22, the right ovary was in the development stage, as manifested by the increasing size of the right ovary and the volume and number of its contained cell. During the degeneration from DE20/GE22 to DP1/GP1, the right ovaries were positioned on the ventromedial surface of the mesonephroi. In comparison to DE20 or GE22, the right ovaries became thinner on P1, and the cells appeared significantly more flattened. Besides, a reduction in cell number and an increase in lacunar channels were also observed, which showed an obvious degradation. In a word, the right ovaries of ducks and geese show a same degradation law of initially undergoing development and followed by subsequent degradation (Fig. 1A and B).

### Identification of DEGs and gene expression profiles during right ovarian degeneration

**Fig. 2:**
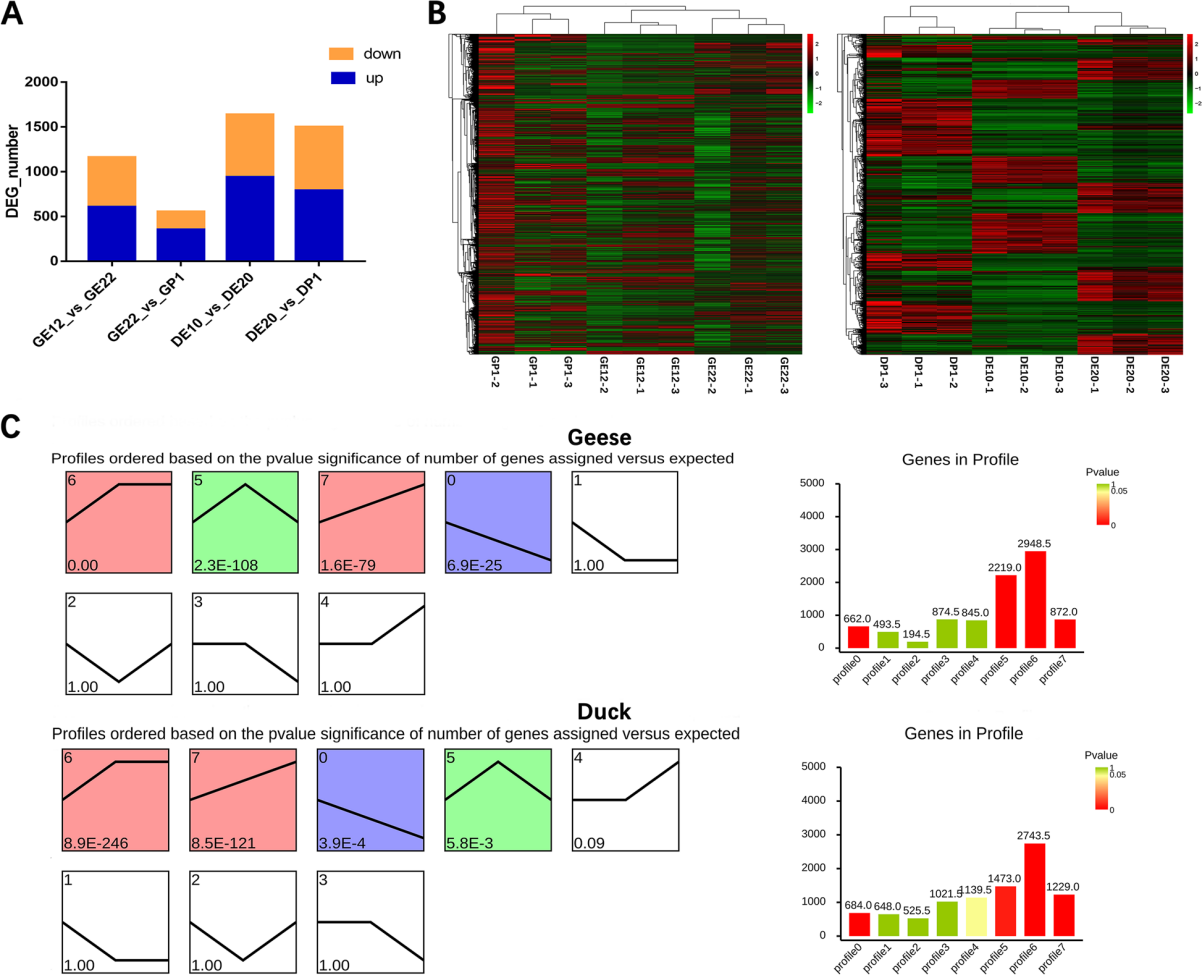
The DEGs during the degeneration of the right ovary. **A** Statistics of the number of up-regulated and down regulated DEGs; **B** The heat map of all DEGs; **C** Degeneration-dependent patterns of genes in the right ovary

As shown in Supplementary Table S1, the average number of clean data obtained from the 18 samples was 219,484,93, with each sample having a Q30 score of not less than 90%. Comparison efficiency of the filtered clean reads to the reference genome was 77.39% for geese and 87.76% for ducks on average (Supplementary Table S2). TPM-based quantitative analysis was employed to analyze quantitative expression changes of identified genes during right ovarian degradation. Log2(foldchange) > 1.5, FDR < 0.05 were used as the condition for screening DEGs. The clustering heatmap in Fig. 2B demonstrated the distinct clustering of the 18 RNA-seq libraries into three clusters corresponding to DE10, DE20, DP1 or GE12, GE22, and GP1 (Fig. 2B). Furthermore, a total of 1161 DEGs were identified between GE12 and GE22, with 606 up-regulated and 555 down-regulated. Additionally, 551 DEGs were identified between GE22 and GP1, including 353 up- and 198 down-regulated DEGs. Similarly, between DE10 and DE20, a total of 1637 DEGs were identified, with 940 up- and 697 down-regulated DEGs.Between DE20 and DP1, 1499 DEGs were identified, of which 789 were up-regulated and 710 were down-regulated (Fig. 2A, Supplementary Table S3). Then a time-course gene expression analysis was further conducted, revealing eight distinct expression patterns. Among these, profile6 exhibited up-regulated between DE10-DE20 or GE12-GE22, with no significant change between DE20-DP1 or GE22-GP1, profile5 showed up regulated between DE10-DE20 or GE12-GE22, down-regulated between DE20-DP1 or GE22-GP1, profile7 displayed up-regulating during both phases were the top three profiles with the largest number of enriched genes (Fig. 2C).

### Identification of key genes related to right ovarian degeneration

**Fig. 3:**
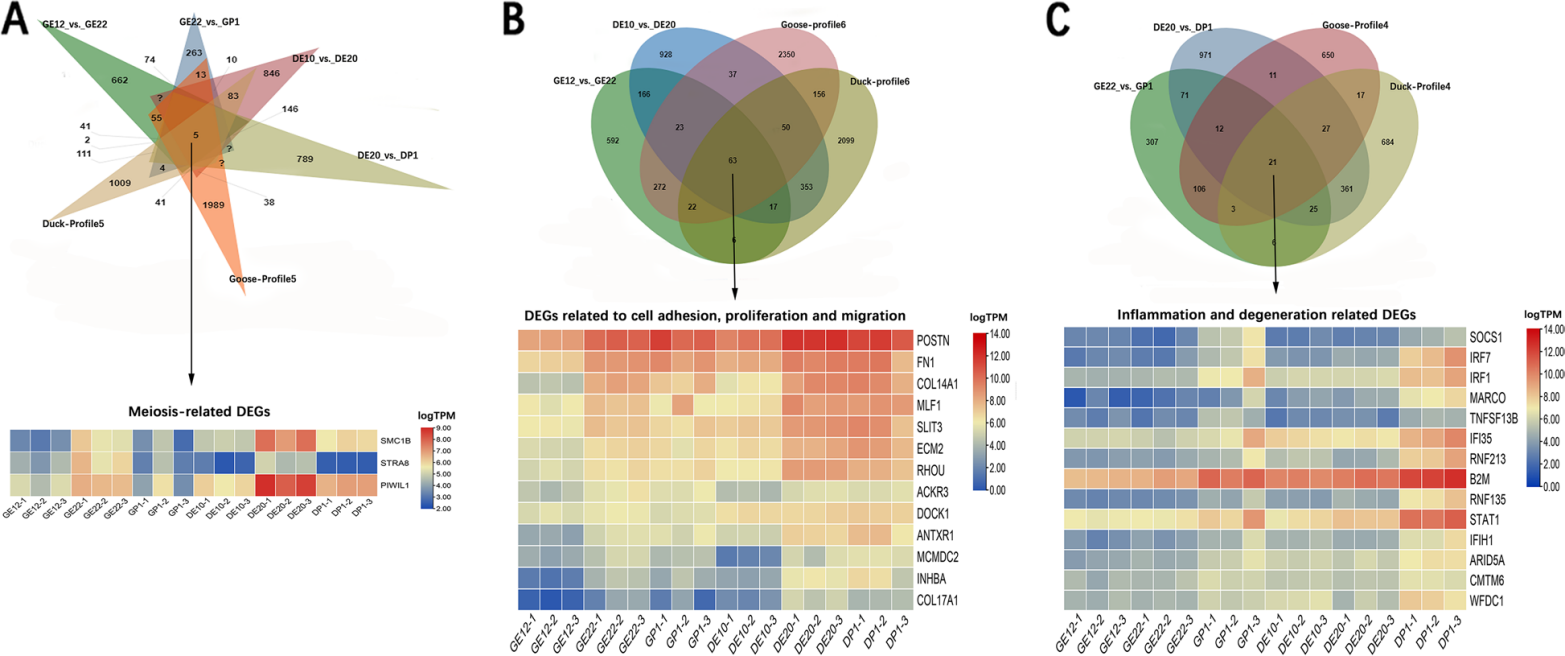
Combined analysis of DEGs and the different gene expression profiles. **A** The venn illustrates the intersections genes between DEGs of DE20_vs._DP1/GE22_vs._GP1 and profile 5 (up), and a corresponding heat map displays the intersections genes related to meiosis (down). **B** The venn illustrates the intersections genes between DEGs of DE10_vs_DE20/ GE12_vs_GE22 and profile6 (up), and a corresponding heat map displays the intersections genes related to cell adhension, migration, meiosis, promotion of proliferation, and inhibition of apoptosis (down). profile 5. **C** The venn illustrates of the intersections genes between DEGs of DE20_vs_DP1/ GE22_vs_GP1 and profile 6 (up), and a corresponding heat map displays the intersections genes related to inflammation, growth inhibition, vascular degenerate promotion (down)

During the degeneration of the right ovary, genes that promote development may show up-regulation during the developmental stage and then either cease up-regulation or down-regulated during the degenerative stage; On the other hand, genes that promote degeneration may exhibit up-regulation during the degenerative stage. Therefore, the genes belonging to profile 5 (up-down) and profile 6 (up-maintain) may be be associated with the the development of the right ovary. Genes belonging to profile 4 (maintain-up) may be related to the degeneration of the right ovary. A combined analysis of the DEGs and the genes with specific expression patterns was conducted to screen the key genes involved in regulating of the development and degeneration of the right ovary. As shown in Fig. 3, firstly, by analyzing the DEGs between DE20_vs._DP1/GE22_vs._GP1 and profile 5, five DEGs were identified. Among these, three genes (SMC1B, PIWIL1, STRA8) were found to be related to meiosis. Secondly, through combined analysis of DEGs between DE10_vs_DE20/ GE12_vs_GE22 and profile 6, a total of 63 DEGs were screened out. Thirteen of these genes were linked to cell adhesion (POSTN, FN1, COL14A1, ECM2, RHOU, ANTXR1, COL17A1), migration (SLIT3, DOCK1), meiosis (MCMDC2), promotion of proliferation/inhibition of apoptosis (MLF1, ACKR3). 21 DEGs were screened by combined analysis of DEGs between DE20_vs_DP1/ GE22_vs_GP1 and profile 4, out of which 12 were related to inflammation (SOCS1, IRF7, IRF1, MARCO, STAT1, TNFSF13B, IFI35, CMTM6, B2M, RNF135, IFIH1, ARID5A). Additionally, the analysis revealed the presence of WFDC1, a gene known for its growth inhibitory activity, and RNF213, a gene implicated in promoting vascular degeneration. These results suggest that the degeneration of the right ovary may be accompanied by the inhibition of meiosis and the activation of inflammation.

### Analysis of common GO terms and KEGG pathways enriched by DEGs in both ducks and geese

To analyze the molecular mechanism underlying right ovarian degeneration, GO and KEGG [28–30] enrichment analysis were performed on DEGs during embryonic degeneration of the right ovary of ducks and geese. The DEGs between DE10_vs._DE20 were significantly enriched in 74 GO terms (Supplementary Figure S2A), and 40 KEGG pathways (Supplementary Figure S3A). DEGs between DE20_vs_DP1 were significantly enriched in 100 GO terms (Supplementary Figure S2B), and 28 KEGG pathways (Supplementary Figure S3B) (Supplementary Table S4). Similarly, DEGs between GE12_vs_GE22 were significantly enriched in 91 GO terms (Supplementary Figure S2C), and 50 KEGG pathways (Supplementary Figure S3C). Subsequently, the DEGs between GE22_vs._GP1 resulted in the enrichment of 123 GO terms and 40 KEGG pathways (Supplementary Figure S2D, S3D) (Supplementary Table S4).

**Fig. 4:**
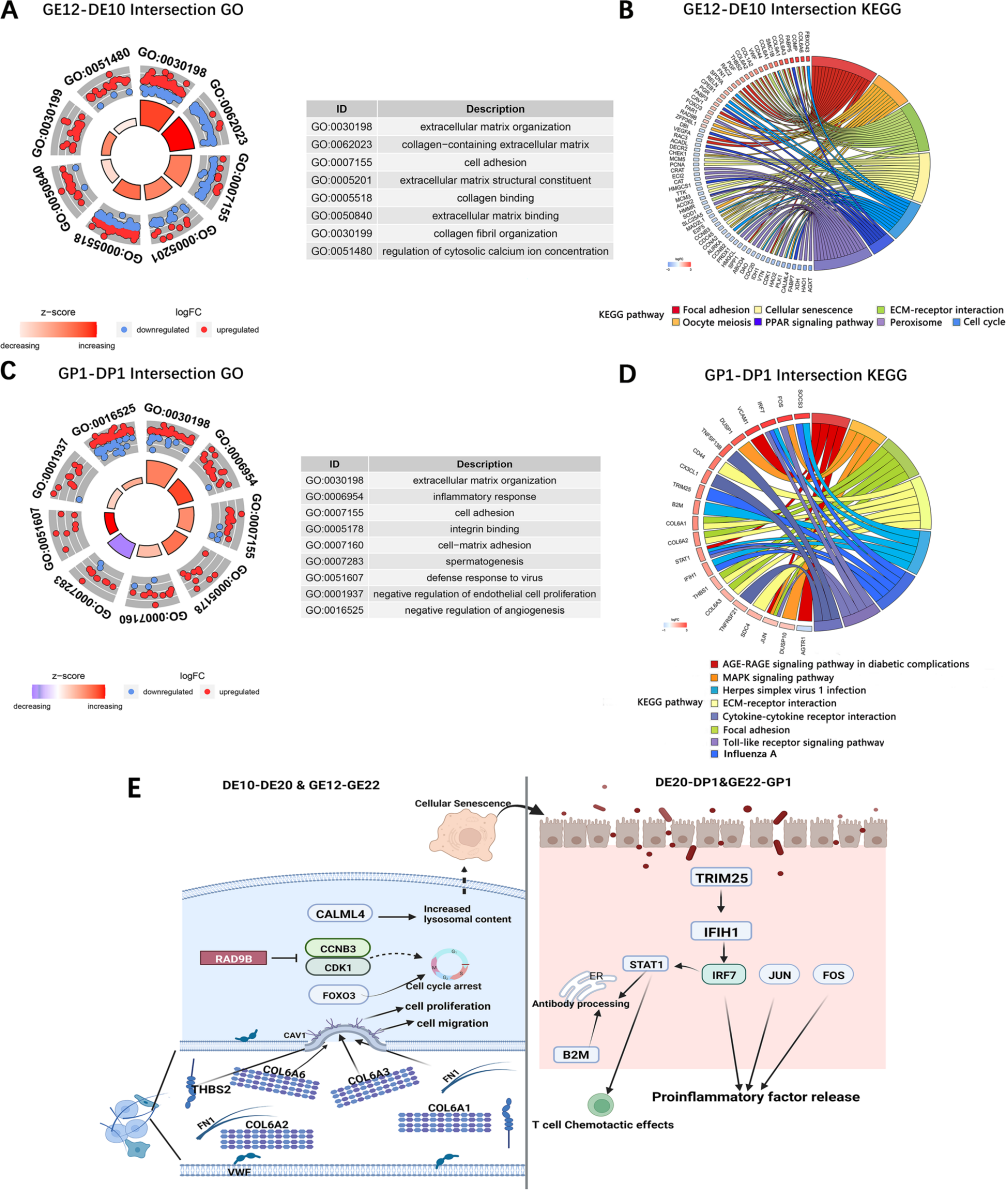
The Intersection of KEGG and GO terms enriched by DEGs in both ducks and geese during right ovary degeneration. **A** The intersection GO terms enriched by DEGs both between DE10 vs. DE20 and GE12 vs. GE22. Each box represent one interaction GO term, red point represent the up-regulated DEGs, blue point represent the down-regulated DEGs; **B** The intersection KEGG pathways enriched by DEGs both between DE10 vs. DE20 and GE12 vs. GE22. Different colors of the lines indicate different KEGG pathways. The squares along the lines represent genes enriched in those pathways, and the color of the squares transitions from red to blue means the log2foldchange score gradually increases from -3 to 3;. **C** The intersection GO terms enriched by DEGs both between DE20 vs. DP1 and GE22 vs. GP1; **D** The intersection KEGG pathways enriched by DEGs both between DE20 vs. DP1 and GE22 vs. GP1; **E** Predicted regulatory mechanism of right ovary degeneration in ducks and geese

According to the section results of the right ovaries of ducks and geese during the embryonic stage up to the first-day post-hatching, the right ovaries of geese and ducks showed a similar degradation pattern. Based on this observation, we further identified the key molecular mechanism involved in the development and degeneration of the right ovary by integrating the results of functional enrichment analysis of DEGs in both ducks and geese. The results revealed that there were 27 GO terms that exhibited significant enrichment in both the GO terms enriched by DEGs between the DE10_vs._DE20 and the GO terms enriched by the DEGs between GE12_vs._GE22, of which mainly including cell adhesion, extractable matrix structural condition, collagen-containing extractable matrix, regulation of cytosolic calcium concentration, collagen fiber organization, extractable matrix organization, extractable matrix binding, Collagen binding (Fig. 4A). In addition, the pathways Focal adhesion, cellular senescence, ECM-receptor interaction, Oocyte meiosis, PPARγ signaling pathway, peroxisome, cell cycle pathways et al. were enriched by DEGs in both ducks and geese (Fig. 4B). While both the DEGs of DE20_vs._DP1 and GE12_vs._GP1 were significantly enriched in 37 GO terms, primarily involving defense response to a virus, inflammatory response, cell adhesion, negative regulation of angiogenesis, extracellular matrix organization, spermatogenesis, integrin binding, negative regulation of endothelial cell proliferation, cell–matrix adhesion (Fig. 4C). Furthermore, several pathways related to inflammation (Herpes simplex virus 1 infection, Influenza A, Toll-like receptor signaling pathway), cell adhesion (ECM-receptor interaction, Focal adhesion), and metabolism (Cysteine, and methionine metabolism, Adipocytokine signaling pathways) were also enriched by both DEGs in ducks and geese (Fig. 4D). These findings align with the functions of the identified key DEGs, suggesting that cell adhesion-related pathways play a crucial role in the development of the right ovary of ducks and geese. The degeneration of the right ovary during the late embryonic stage in both species may be related to the activation of inflammatory response. According to the pathway map in KEGG database and the shared DEGs between ducks and geese, during the developmental stages of the right ovary, collagen genes (*COL6A1, COL6A2, COL6A3,* and *COL6A6*) and fibronectin genes (*FN1*), thrombospondin gene (*THBS2*) are implicated in regulating cell proliferation and migration through scaffolding protein within caveolar membranes (*CAV1*). On the other hand, during the degeneration of the right ovary, certain genes such as *RAD9B, FOXO3,* and *CALML4* are involved in inducing cell senescence. This process is followed by the up-regulation of inflammation-related genes including *STAT1, IRF7, JUN,* and *FOS*. Consequently, the release of inflammatory factors lead to the activation of inflammatory responses. This inflammatory cascade ultimately contributes to the progressive degeneration of the right ovary (Fig. 4E).

### Analysis of specific DEGs and specific enrichment pathways in ducks and geese

Among the DEGs between DE10_vs._DE20 and GE12_vs._GE22, a total of 1368 genes were specifically differentially expressed in the right ovary of ducks. These genes exhibited significant enrichment in 16 KEGG pathways and 30 GO terms. Additionally, 892 genes were specifically differentially expressed only in the right ovary of geese, and they showed significant enrichment in 45 KEGG pathways and 73 GO terms (Supplementary Table S5). Furthermore, DEGs between DE20_vs._DP1 and GE22_vs._GP1, 1368 genes were specifically differentially expressed in the right ovary of ducks. These genes were significantly enriched in 20 KEGG pathways and 61 GO terms. On the other hand, 422 genes were specifically differentially expressed only in the right ovary of geese, and they exhibited significant enrichment in 33 KEGG pathways and 69 GO terms (Supplementary Table S5).

**Fig. 5:**
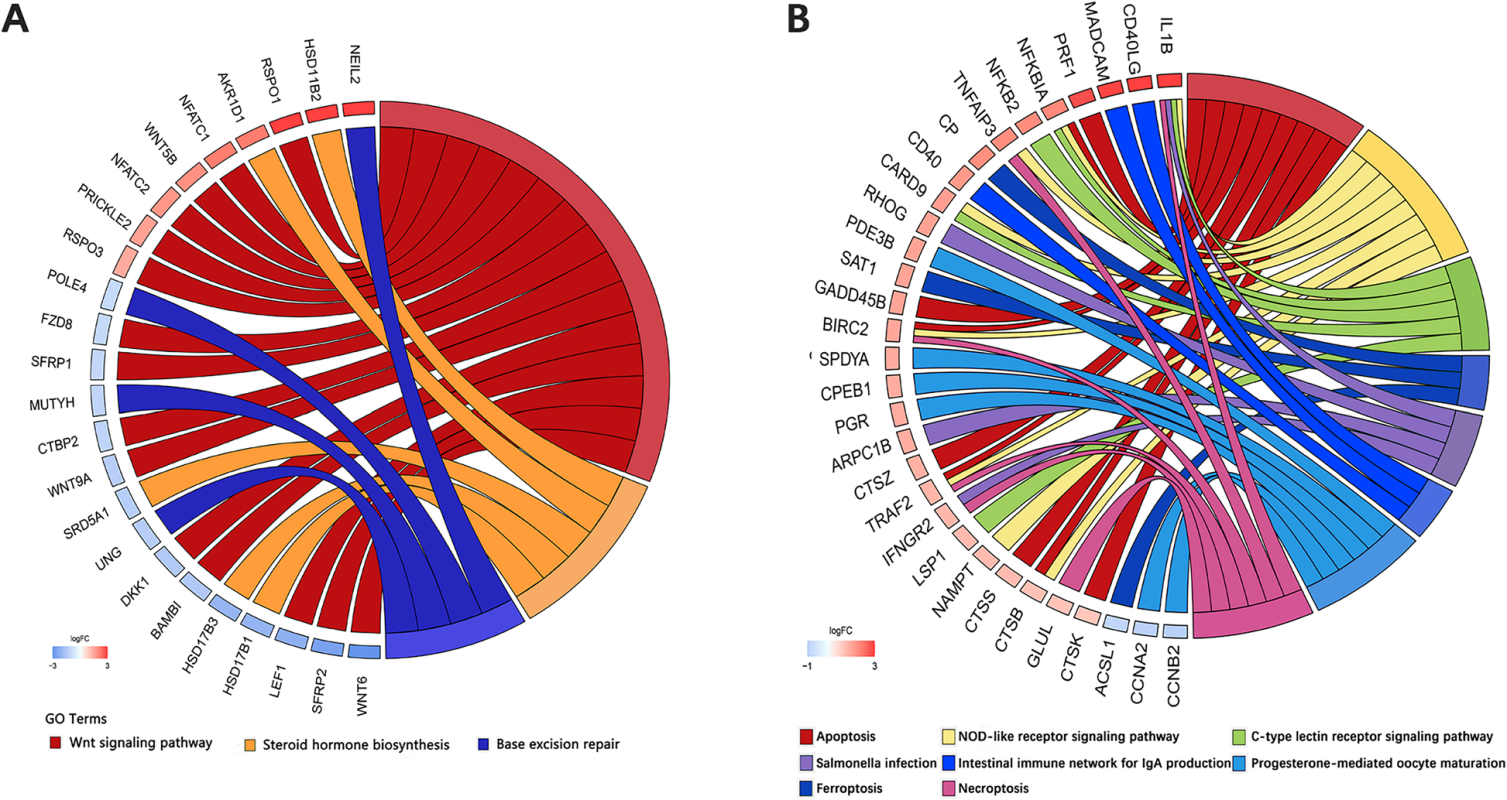
The specificity KEGG pathway enriched by specific DEGs. **A** The chordal graph of the specificity KEGG pathway enriched by specific DEGs of ducks; **B** The chordal graph of the specificity KEGG pathway enriched by specific DEGs geese. Different colors of the lines indicate different KEGG pathways. The squares along the lines represent genes enriched in those pathways, and the color of the squares transitions from red to blue means the log2foldchange score gradually increases from -3 to 3

During the early stage of embryonic development, the DEGs in the right ovary of the duck exhibited specifical enrichmen in the Wnt signaling pathway, Steroid hormone biosynthesis, and Base excision repair pathway (Fig. 5A). In contrast, the specifically DEGs of the goose’s right ovary were found to be specifically enriched in the other inflammation and apoptosis-related pathways, including NOD-like receptor signaling pathway, C-type lectin receptor signaling pathway, Salmonella infection, apoptosis, ferroptosis, etc. (Fig. 5B). These findings suggested that, at the embryonic stage, the degeneration of the right ovary in ducks may be slower than that in geese.

The genes specifically expressed in ducks and geese's right ovaries were further analyzed separately. In the right ovary of ducks, 9309 genes were found to specifically expressed, out of which 595 genes were differentially expressed. The top 20 annotated genes with the highest expression level were predominantly associated with inflammation (*TRAIP, NFIC, FAM65B, IL1B, TAP2, CD3D, TAGAP, BIRC2*) and cell proliferation (*CCNB1, INIP, NASP, DYNAP, CYR61*) (Table 2). Similarly, in the right ovary of geese, a total of 12,877 genes were specifically expressed, and 1213 of them displayed differential expression. Among the top 20 annotated genes with the highest expression level, there are 5 inflammation-related genes (*IFITM2, CEBPD, DHX58, IFITM1, CCL19*), 3 cell proliferation and anti-apoptosis-related genes (*TCIM, CCN1, VEGFD, PHLDA1*), and 3 negative regulators of inflammatory response *CD24*, *TSC22D3*, and *CCN3* (Table 3). Among the genes specifically expressed in the right ovary of ducks, there was a notable up-regulation of genes associated with promoting proliferation, anti-apoptosis, and anti-inflammatory in the DE20 vs. DP1 comparison. On the other hand, among the geese-specific genes, the genes that exhibited significant up-regulation in the GE22 vs. GP1 comparison were primarily inflammation-related. These findings indicate that there might be inherent differences in the degree of degeneration of the right ovary between ducks and geese during the embryonic stage.

**Table 2:** The top 20 specifically expressed DEGs with the highest expression level of ducks

DEGs	TPM			log2FoldChange		Function
	DE10	DE20	DP1	DE10 vs. DE20	DE20 vs. DP1	
IFITM2	2100.943	3045.972	6207.41	NA	1.07	IFN-induced antiviral protein which inhibits the entry of viruses to the host cell cytoplasm
TAGLN	56.49037	122.3444	2878.295	NA	4.59	May contribute to replicative senescence
CD24	545.7143	469.0568	1696.371	NA	1.9	May have a pivotal role in cell differentiation of different cell types
TSC22D3	164.6922	443.8537	1011.774	1.28	1.23	In macrophages, plays a role in the anti-inflammatory and immunosuppressive
HSD3B1	465.1404	108.1778	883.4034	-2.25	NA	A bifunctional enzyme responsible for the essential step in steroid hormone biosynthesis
CCN2	195.2611	250.0084	749.4131	NA	1.63	Major connective tissue mitoattractant secreted by vascular endothelial cells
HSD17B1	1912.734	2678.402	611.7204	NA	-2.09	Converts estrone (E1) to a more potent estrogen, 17beta-estradiol (E2)
CEBPD	60.38073	136.4143	504.8173	1.02	1.93	Important transcription factor regulating the expression of genes involved in immune and inflammatory responses
CCN3	38.51077	521.3119	426.4187	3.6	NA	Plays a role as negative regulator of endothelial pro-inflammatory activation
RPRM	38.67574	42.64927	331.3235	NA	3.01	May be involved in the regulation of p53-dependent G2 arrest of the cell cycle
TCIM	65.35551	106.6691	327.5122	NA	1.66	Seems to be involved in the regulation of cell growth an differentiation
CCN1	61.10661	59.30382	317.1072	NA	2.46	Promotes cell proliferation, chemotaxis, angiogenesis and cell adhesion
DHX58	4.360706	9.631986	282.1522	NA	4.92	Acts as a regulator of DDX58/RIG-I and IFIH1/MDA5 mediated antiviral signaling
PHLDA1	155.7158	132.2777	272.521	NA	1.09	May be involved in regulation of anti-apoptotic effects of IGF1
VEGFD	10.149	66.61808	266.3692	2.54	2.04	Growth factor active in angiogenesis,and endothelial cell growth, stimulating their proliferation and migration
IFITM1	18.97657	7.089193	235.5643	NA	5.12	IFN-induced antiviral protein which inhibits the entry of viruses to the host cell cytoplasm
CADM3	37.01282	57.95032	206.8719	NA	1.89	Involved in the cell–cell adhesion
CCN4	21.33298	92.77809	193.7107	1.97	NA	Downstream regulator in the Wnt/Frizzled-signaling pathway
ECRG4	1644.404	441.6095	189.8815	-2.05	-1.17	Induce senescence
CCL19	5.769264	4.651705	47.30699	NA	3.49	May play a role not only in inflammatory

**Table 3:** The top 20 specifically expressed DEGs with the highest expression level of geese

DEGs	TPM			log2FoldChange		Function
	GE12	GE22	GP1	GE12 vs. GE22	GE22 vs. GP1	
CCNB1	240.7181	43.87955	44.18241	-2.6615	NA	Essential for the control of the cell cycle at the G2/M (mitosis) transition
LHCGR	35.35759	13.23799	8.143971	-1.65383	NA	Receptor for lutropin-choriogonadotropic hormone
FAM64A	56.9678	24.29569	21.03097	-1.40873	NA	During mitosis, may play a role in the control of metaphase-to-anaphase transition
INIP	145.4371	71.40666	41.71304	-1.20884	NA	promote DNA repair and G2/M checkpoint
TRAIP	67.33086	34.48606	36.02288	-1.14766	NA	Also acts as a negative regulator of innate immune signaling
NASP	137.2849	75.82193	56.73469	-1.04428	NA	Required for DNA replication, normal cell cycle progression and cell proliferation
NFIC	27.95951	77.29887	96.66304	1.270449	NA	Recognizes and binds the palindromic sequence 5'-TTGGCNNNNNGCCAA-3' present in viral
FAM65B	4.179413	20.04363	50.98591	2.102482	NA	Plays several roles in the regulation of myoblast and hair cell differentiation, lymphocyte T proliferation
DCSTAMP	0.041356	6.136462	22.44233	4.839821	NA	Involved in the regulation of neutrophil polarization, chemotaxis and adhesion
DYNAP	0.072471	7.613666	4.302598	5.116443	NA	Plays a role in the regulation of cell proliferation
IL1B	0.085364	0.247621	5.493098	NA	4.996311	Potent proinflammatory cytokine
IL2RA	0.413104	2.13566	27.00625	NA	3.693057	The receptor is involved in the regulation of immune tolerance
TNXB	0.402312	2.31242	24.09526	NA	3.470337	Appears to mediate interactions between cells and the extracellular matrix
TAP2	7.882012	8.763727	64.71774	NA	3.000475	ABC transporter associated with antigen processing
CD3D	13.67938	24.38414	128.3267	NA	2.597159	plays an essential role in adaptive immune response
CYR61	31.47393	38.49783	161.3469	NA	2.221431	Promotes cell proliferation, chemotaxis, angiogenesis and cell adhesion
TAGAP	2.520332	16.21987	51.50274	2.609794	1.807563	May function as a GTPase-activating protein and may play important roles during T-cell activation
GADD45B	78.1767	79.7639	214.394	NA	1.560371	Involved in the regulation of growth and apoptosis
BIRC2	53.30691	51.13248	131.5747	NA	1.52566	Multi-functional protein which regulates not only caspases and apoptosis, but also modulates inflammatory signaling and immunity
ATAD2	42.73945	100.3824	41.14164	1.04478	-1.1552	May induce the expression of a subset of estradiol target genes, such as CCND1, MYC and E2F1
HSF2BP	11.93186	31.25799	7.307502	NA	-2.01293	Meiotic recombination factor component of recombination bridges involved in meiotic double-strand break repair

### Comparison of left ovary development between geese with rapid and slow rates of right ovary degeneration

**Fig. 6:**
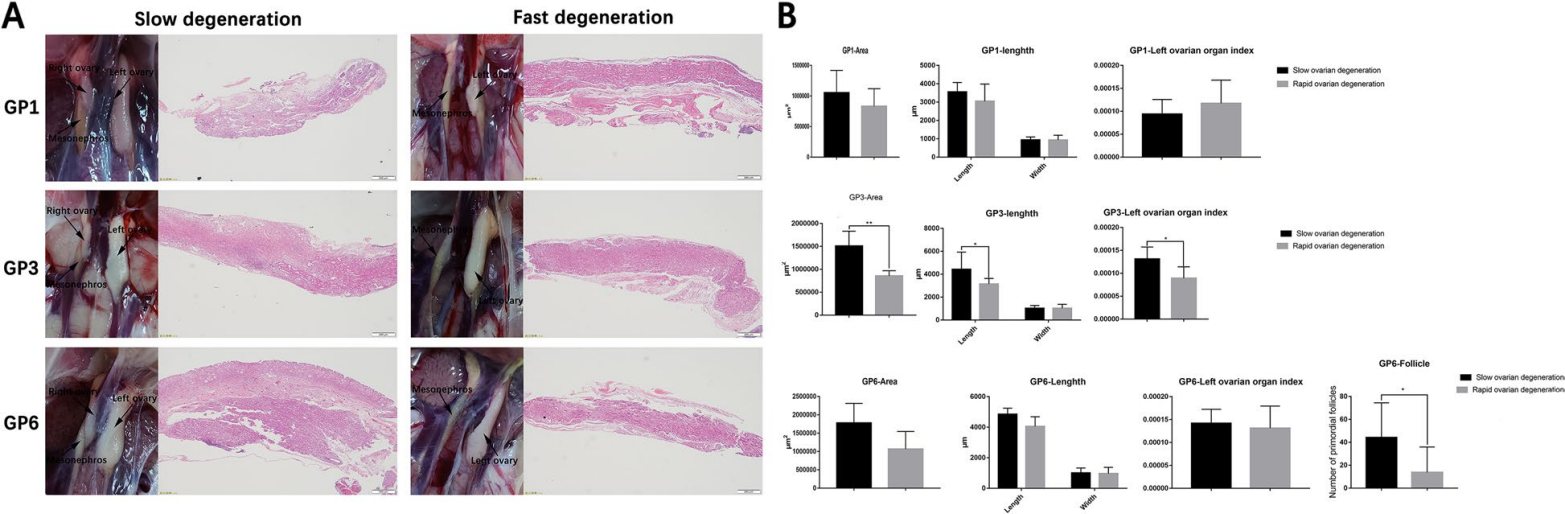
Difference analysis of left ovary between slow and fast degenerated group geese. **A** Anatomy and histology structure of the right ovary in fast and slow degenerated group. **B** Comparison of left ovarian organ index, area, long diameter, short diameter and number of primordial follicles on the GP6 between the fast and slow degeneration group. The middle kidney, left ovary and right ovary (if exists) are indicated in the anatomical picture. Since the right ovary was not observed in the geese of the rapid degeneration group, the structure in the section photos was the structure of the middle kidney

After hatching, there is significant variation in the degree of degeneration observed in the right ovary of geese and ducks. Some individuals show minimal degeneration. with tight structures and no apparent signs of degeneration. In constract, others individuals exhibit extensive degeneration, characterized by the presence of numerous lacunar channels. This variability in degeneration is particularly pronounced in geese. In particular, it is observed that from the 6th day to the 13th day after hatching, certain geese display a distinct pattern where the right ovary does not undergo degeneration. Instead, it maintains a structure similar to that of the left ovary, with the development of a cortical layer and primordial follicles (Supplementary Figure S1). Therefore, according to the degeneration of the right ovary of geese observed in the anatomy and histological section, we identify geese with rapid (Group R) and slow (Group S) rates of right ovary degeneration (Fig. 6A). We compared the organ index, ovarian area, length and width of the left ovary at different time points ( GP1, GP3, and GP6), as well as the number of primordial follicles on the GP6, between the two groups. The results showed that on the GP1, there were no significant differences in the area, long and short diameter, and organ index of the left ovary between the two groups. However, on GP3, geese in Group S exhibited significantly higher values in terms of the area, long and short diameter, and organ index of the left ovary compared to group R (P < 0.05). On GP6, the geese in Group S also displayed higher values for the area, length, diameter, and organ index of the left ovarycompared to group R, but the difference was not statistically significant. Importantly, the number of primordial follicles contained in the left ovary of geese in group S was significantly higher than that in group R (*P* < 0.05) (Fig. 6B). These results provide further evidence to support the existence of a relationship between the degeneration of the right ovary and the development of the left ovary.

Among the DEGs identified through sequencing, one notable gene is *INHBA*, which is involved in the regulation of pituitary hypothalamic gonadotropin secretion. In the development stage of the right ovary in ducks and geese, *INHBA* is up-regulated in the development stage, but its up-regulation ceases in the degradation stage. Additionally, the genes of duck profile 6 (Supplementary Table S6) and goose profile 7 (Supplementary Table S7) were observed significantly enriched in the GnRH signaling pathway. These findings indicate that prior to the degeneration of the right ovary, it may interact with the hypothalamic-pituitary axis to regulate the secretion of gonadotropins and subsequently affecting the development of the left ovary.

### Validation of Expression Profiles of DEGs

**Fig. 7:**
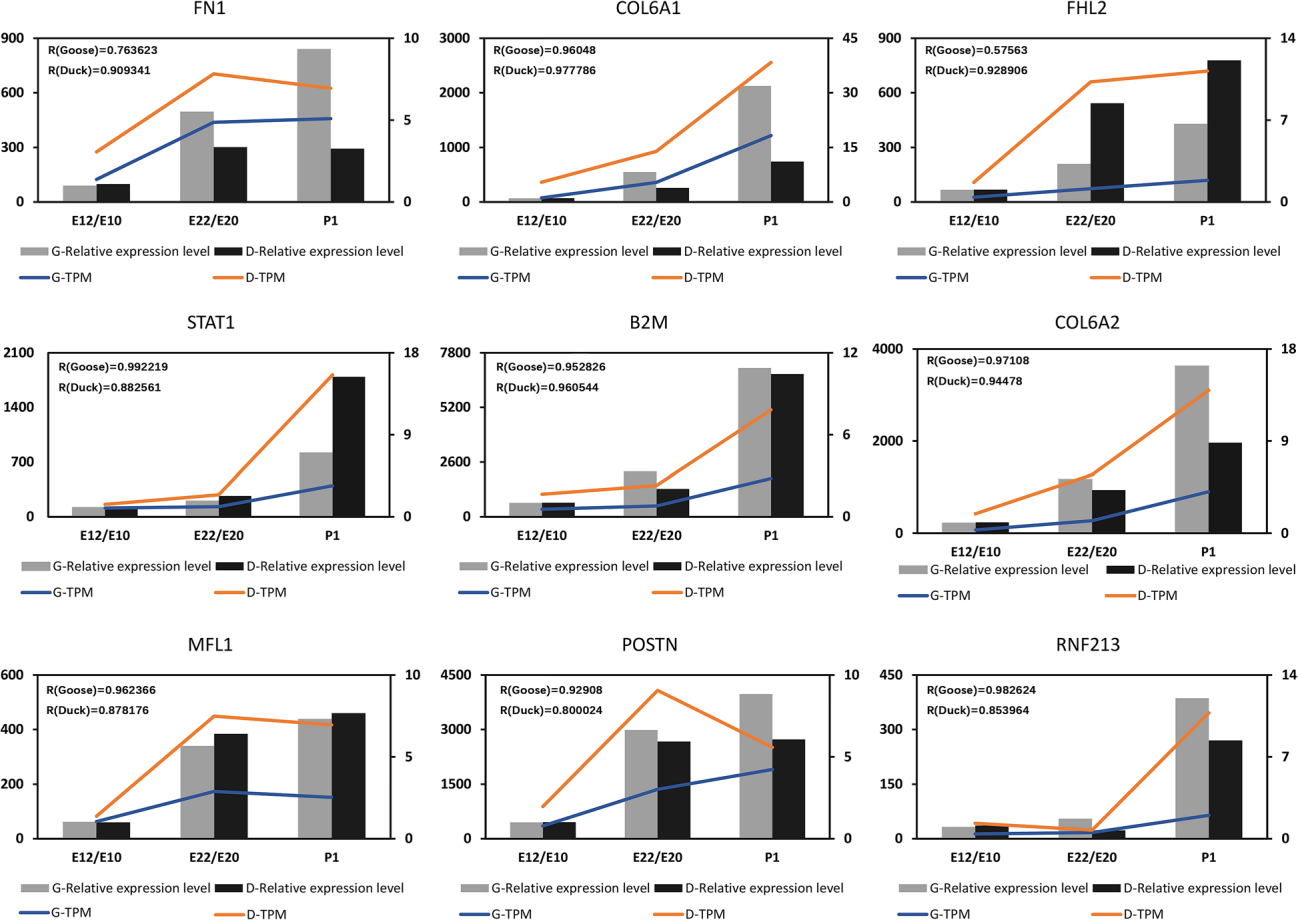
Verification of expression patterns of DEGs. The blue line represents the TPM results obtained by goose RNA-seq, and the orange line represents the TPM results obtained by duck RNA-seq. The gray column represents the results of relative expression in geese detected by qRT-PCR, and the black column represents the results of relative expression in duck detected by duck qRT-PCR

To validate the expression profiles of selected DEGs involved in various biological processes, including cell adhesion (*FN1, COL6A1, COL6A2*), inflammation (*STAT1, B2M*), proliferation (*POSTN*), anti-apoptotic (*MFL1*), meiosis (*FHL2*), and vessel regression (*RNF213*), qPCR was performed using the right ovary of geese and ducks. As shown in Fig. 7, except for *POSTN*, the expression of almost the other eight selected DEGs determined by qRT-PCR displayed changes in the same direction as those identified through RNA-seq. This those identified in the expression patterns of these eight genes corroborated the reliability of the RNA-Seq data.

## Discussion

In avian species, the embryonic ovaries develop asymmetrically, whereby the right ovary gradually degenerates [31]. Although limited information is currently available on the degeneration of the avian right ovary, particularly regarding the histological changes and gene expression patterns during this degenerative progress in ducks and geese. Our result indicate a significant increase in both the volume and number of cells in the right ovary during the early embryonic development (Ducks: DE10-DE20, Geese: GE12-GE22). Subsequently (Ducks: DE20-DP1, Geese: GE22-GP1), the structure of the right ovary becomes loose. This is evidenced by the better development of lacunar channels, the flattening of cells, and a significant reduction in cell number. González-Morán analyzed the histological changes in the right ovary of white Laihang chicken from the embryonic day eight to the age of 1 day to 4th week after hatching. Their stereological results revealed that in the right ovary, there is an initial increase in blood vessels, lacunar channels, and interstitial spaces until one day after hatching, followed by regression and subsequent, decrease in these structures [8]. These findings is consistent with our results, suggesting a similar pattern of initial development followed by gradual degeneration in the right ovary. This further indicates that the right ovaries of poultry exhibit a degenerative pattern of first development and then degeneration.

Cell adhesion pathways appear to play a crucial role in the development of the right ovary in ducks and geese. The functional annotation of DEGs identified according to gene expression profiling indicates that several DEGs (*POSTN *[32], *FN1 *[33, 34], *COL14A1 *[35], *ECM2 *[36], *RHOU *[37], *ANTXR1 *[38], *COL17A1 *[39]) up-regulated during the developmental stage of the right ovary are primarily associated with cell adhesion progress. Furthermore, significant enrichment of DEGs of duck and goose during the developmental stage of the right ovary were observed in cell adhesion-related pathways, including ECM-receptor interaction, Focal adhesion. Extracellular matrices (ECMs) contain collagenous and noncollagenous glycoproteins, glycosaminoglycans, proteoglycans, and associated molecules that make up the metazoan matrisoma [40]. ECM serves multiple roles beyond being a substrate for tissue morphogenesis and providing support and flexibility to mature tissues. It also acts as an epigenetic informational entity by transducing and integrating intracellular signals through specific cell surface receptors. Consequently, the interaction between ECM and its receptor has a profound influence on various cellular processes, including growth, differentiation, migration, and survival [41, 42]. In the case of ducks and geese, DEGs enriched in the ECM-receptor interaction, Focal adhesion pathways are primarily collagen-related genes such as *COL6A3, COL6A1, COL6A2*. The collagen superfamily of proteins, which includes these genes, plays a crucial role in formingthe extracellular matrix, basement membranes, other structures of the extracellular matrix, and maintaining the integrity of various tissues. In addition, collagen proteins also serve several other important functions [43, 44]. Furthermore, emerging evidence has demonstrated that *COL6A1* is included in cell migration, differentiation, embryonic development, and maintenance of cell stemness [45, 46]. In conclusion, the early development of the right ovary in ducks and geese may rely on cell adhesion involving collagen genes *COL6A3*, *COL6A1,* and *COL6A2*. Subsequently, the progressive degeneration of the right ovary appears to involve the activation of the inflammatory response. This is supported by the up-regulation of inflammation-related genes during the degenerative stage. Furthermore, and both the DEGs between DE10 vs. DE20 and GE12 vs. GE22 were significantly enriched in inflammation-related pathways, including Herpes simplex virus 1 infection, Influenza A, and Toll-like receptor signaling pathway. Inflammation is a fundamental and prominent protective response in organism, tightly interconnected with innate immunity. Many inflammatory mediators are a part of innate immunity system that exists all multicellular organisms [47]. Aseptic inflammation involves essential processes such as neutrophil phagocytosis, pathogens killing,generation of reactive oxygen species (ROS) and proinflammatory factors, and the release of neutrophil extracellular traps (NETs) or apoptosis [48, 49]. Subsequently, dying cells are phagocytized by macrophages, known as efferocytosis [48]. Inflammation is closely related to the impairment of ovarian function. Emerging evidence suggests that polycystic ovary syndrome is a proinflammatory state, and inflammation underpins the development of metabolic abnormalities and ovarian dysfunction [50]. These studies collectively indicate that during late embryonic development, the activation of the inflammatory response leads to apoptosis of, which are then cleared through phagocytosis. This process resulte in progressive loosening of the right ovarian structure and gradual degeneration. Moreover, both the DEGs in ducks and geese between DE20 vs. DP1 and GE22 vs. GP1 were found to be enriched in the cellular senescence pathway. Cellular senescence refers to a stable cell cycle arrest that occurs in diploid cells, limiting their proliferative lifespan [51]. Senescent cells exhibit a complex pro-inflammatory response called senescence-associated secretory phenotype (SASP) [52]. This suggests that cellular senescence of the right ovary during early embryonic development in ducks and geese may trigger an inflammatory response in the late embryonic right ovary, subsequently promoting its degeneration. Furthermore, Shaikat, A.H. and his colleagues investigate gene expression profiling in the embryonic chicken ovary during asymmetric development [20]. The KEGG enrichment analysis of the DEGs between CE12 vs. CP1 in the chicken right ovary were also significantly enriched in inflammation-related pathways (C-type lectin receptor signaling pathway, NOD-like receptor signaling pathway etc.) (Supplementary Table S8). This finding provides further evidence that the occurrence of an inflammatory response is a crucial molecular event in the degeneration of the right ovary in avian species. In coclusion, the development of the right ovary is mainly regulated by the *COL6A3*, *COL6A1,* and *COL6A2* mediated cell adhesion pathway, while the degeneration of right ovary mainly regulated by inflammation related pathways.

Our results also reveal significant variations in the degree of degeneration of the right ovary among different individuals after hatching. During female gonadal sex differentiation, both the left and right medulla synthesize estrogen, which is required for ovarian developmentand play both paracrine and autocrine role [10, 53]. This suggests that the right ovary may be involved in regulating the development of the left ovary. By observing the left ovaries of geese varing degree of right ovary degeneration on GP1, GP3, and GP6, we observed that the geese with slower degeneration of the right ovary exhibited larger left ovaries on GP3 and a higher number of primordial follicles on the GP6. This finding further supports the hypothesis that degeneration of the right ovary may impact the development of the left ovary. Analysis of DEGs revealed that Inhibin subunit beta A (*INHBA*) is up-regulated during the development stage, and ceases its up-regulation during the degradation stage of the right ovary in ducks and geese. *INHBA* encodes the bA-subunit of certain activin and inhibin complexes [54]. Inhibins have been known to enhance the production and secretion of follicle-stimulating hormone (FSH) by anterior pituitary cells, which play a crucial role in folliculogenesis, oocyte maturation, and embryo development [55]. In addition, the genes that exhibited continuous up-regulation during the degeneration of the right ovary in geese were also significantly enriched in the GnRH signaling pathway. This further indicates that the right ovary may influence the development of the left ovary through the hypothalamic-pituitary-ovarian axis before its degeneration in geese.

## Conclusions

**Fig. 8:**
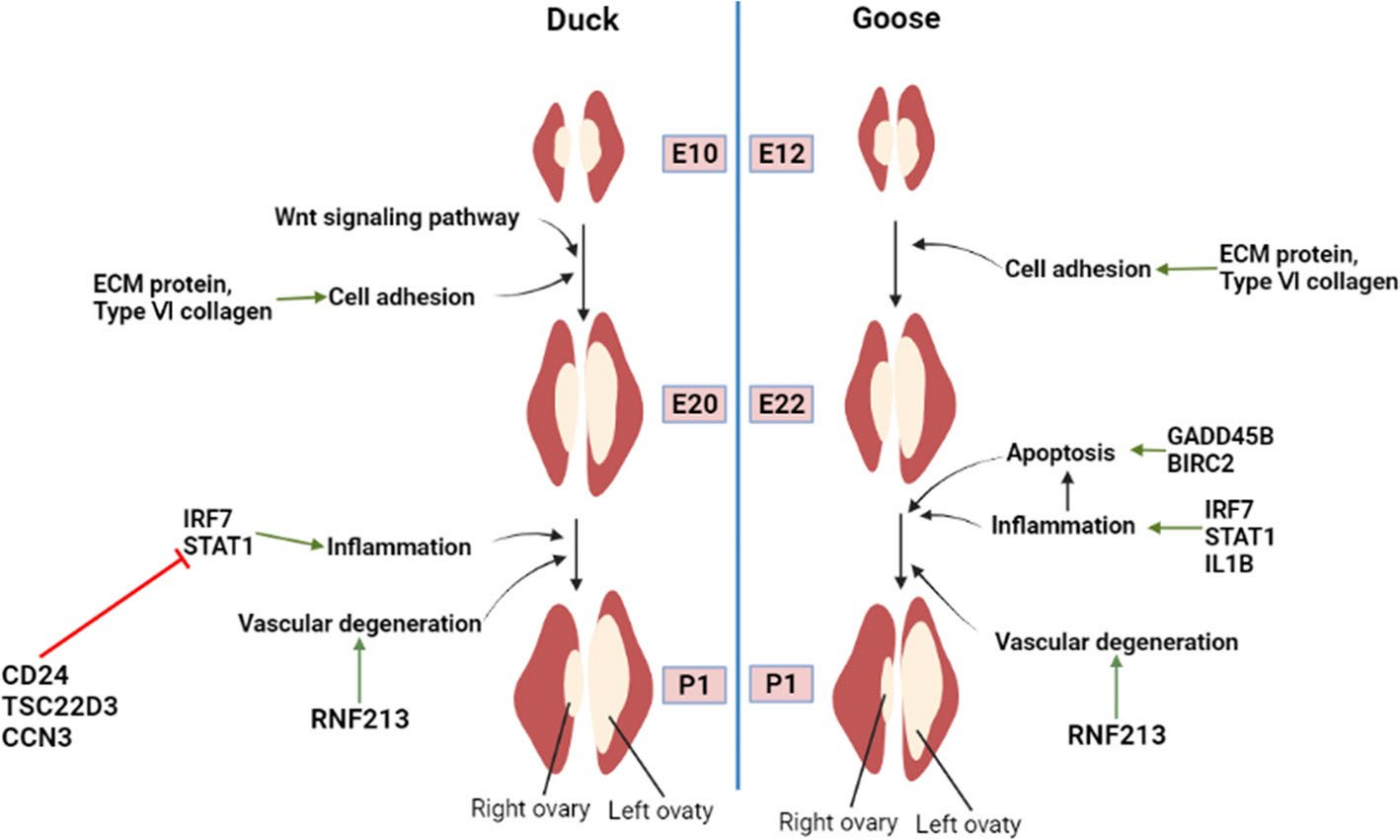
The molecular mechanisms of right ovary degeneration in ducks and geese

In conclusion, the right ovary of ducks and geese undergoes initial development during the embryonic stage, followed by degeneration in the late embryonic stage. Transcriptome sequencing results revealed that the early development of the right ovary of ducks and geese may involve processes such as cell proliferation and migration regulated by ECM-receptor interaction and the Focal adhesion pathway. Conversely, its degradation appears to be associated with cellular aging and the activation of the inflammatory response (Fig. 8). Moreover, We further found that geese with slower degeneration of the right ovaries exhibit faster development in the left ovary. Transcriptome dynamic analysis revealed continuous up-regulation of certain genes enriched in the GnRH signaling pathway in the right ovary during the embryonic stage. This suggests that the right ovary may influence the development of the left ovary through interaction with hypothalamic-pituitary before complete degeneration.

## Supplementary Information

**Additional file 1:** **Supplementary Figure S1. **Comparison ofthe number of primordial follicles in the left ovary of individuals withdifferent degeneration of the right ovary on the 6th and 12th, 13th days afterposting; **Supplementary Figure S2.** The top ten Biologicalprocesses, Cellular components, and Molecular function terms enriched by DEGsbetween DE10 vs. DE20 (A), DE20 vs. DP1 (B), GE12 vs. GE22 (C), GE22 vs. GP1(D);** Supplementary Figure S3.** The top ten KEGG pathways enriched by DEGsbetween DE10 vs. DE20 (A), DE20 vs. DP1 (B), GE12 vs. GE22 (C), GE22 vs. GP1(D); **Supplementary Table S1.** Overview of sequencing data; **Supplementary Table S2. **Mapping rate of RNA-Seq data to the goose reference genome; **Supplementary Table S3.** All DEGs list; **Supplementary Table S4. **GO and KEGGenrichment analysis results of DEGs. **Supplementary Table S5.** GO and KEGG enrichment results of duck- and goose-specific DEGs, respectively. **Supplementary Table S6. **GO and KEGG enrichment results of genes with different profiles in duck. **Supplementary Table S7. **GO and KEGG enrichment results of geneswith different profiles in goose.** Supplementary Table S8.** DEGs and theenrichment results of GO and KEGG during the degeneration of chicken rightovary.

## Data Availability

All transcriptome sequencing reads are openly available in NCBI SRA at https://submit.ncbi.nlm.nih.gov/, reference number PRJNA821376.
