# Supplementary material for: Exploring right ovary degeneration in duck and goose embryos by histology and transcriptome dynamics analysis
Source: BMC Genomics. 2023 Jul 10;24:389. doi: 10.1186/s12864-023-09493-0 (PMC10332064; doi:10.1186/s12864-023-09493-0)
Supplement: Supplementary file 1 — Additional file 1: Supplementary Figure S1. Comparison ofthe number of primordial follicles in the left ovary of individuals withdifferent degeneration of the right ovary on the 6th and 12th, 13th days afterposting; Supplementary Figure S2. The top ten Biologicalprocesses, Cellular components, and Molecular function terms enriched by DEGsbetween DE10 vs. DE20 (A), DE20 vs. DP1 (B), GE12 vs. GE22 (C), GE22 vs. GP1(D); Supplementary Figure S3. The top ten KEGG pathways enriched by DEGsbetween DE10 vs. DE20 (A), DE20 vs. DP1 (B), GE12 vs. GE22 (C), GE22 vs. GP1(D); Supplementary Table S1. Overview of sequencing data; Supplementary Table S2. Mapping rate of RNA-Seq data to the goose reference genome; Supplementary Table S3. All DEGs list; Supplementary Table S4. GO and KEGGenrichment analysis results of DEGs. Supplementary Table S5. GO and KEGG enrichment results of duck- and goose-specific DEGs, respectively. Supplementary Table S6. GO and KEGG enrichment results of genes with different profiles in duck. Supplementary Table S7. GO and KEGG enrichment results of geneswith different profiles in goose. Supplementary Table S8. DEGs and theenrichment results of GO and KEGG during the degeneration of chicken rightovary. [file 12864_2023_9493_MOESM1_ESM.zip › Supplementary Table S1.docx]

**Table S1** **Overview of sequencing data**

| **Sample** | **Raw Reads** | **Clean Reads** | **Raw Base(G)** | **Clean Base(G)** | **Effective Rate(%)** | **Error Rate(%)** | **Q20(%)** | **Q30(%)** | **GC Content(%)** |
| --- | --- | --- | --- | --- | --- | --- | --- | --- | --- |
| GE22-1 | 23,635,841 | 22,491,524 | 7.09 | 6.75 | 95.16 | 0.03 | 97.75 | 93.75 | 46.95 |
| GE22-2 | 20,224,838 | 19,403,792 | 6.07 | 5.82 | 95.94 | 0.03 | 97.17 | 92.42 | 46.58 |
| GE22-3 | 23,181,854 | 22,398,212 | 6.95 | 6.72 | 96.62 | 0.03 | 97.53 | 93.24 | 46.74 |
| GE12-1 | 22,385,101 | 21,332,587 | 6.72 | 6.4 | 95.3 | 0.03 | 97.34 | 92.78 | 46.69 |
| GE12-2 | 20,091,628 | 19,228,089 | 6.03 | 5.77 | 95.7 | 0.03 | 97.06 | 92.1 | 47.45 |
| GE12-3 | 23,372,367 | 22,352,710 | 7.01 | 6.71 | 95.64 | 0.03 | 97.33 | 92.81 | 47.63 |
| GP1-1 | 22,892,212 | 22,018,017 | 6.87 | 6.61 | 96.18 | 0.03 | 97.4 | 92.94 | 48.36 |
| GP1-2 | 26,976,611 | 25,892,001 | 8.09 | 7.77 | 95.98 | 0.03 | 97.02 | 91.9 | 47.95 |
| GP1-3 | 24,790,075 | 23,736,210 | 7.44 | 7.12 | 95.75 | 0.03 | 97 | 92.13 | 48.82 |
| DE10-1 | 21,929,492 | 21,076,631 | 6.58 | 6.32 | 96.11 | 0.03 | 97.24 | 92.66 | 48.61 |
| DE10-2 | 21,768,007 | 20,740,368 | 6.53 | 6.22 | 95.28 | 0.03 | 97.25 | 92.72 | 48.42 |
| DE10-3 | 20,946,416 | 20,210,845 | 6.28 | 6.06 | 96.49 | 0.03 | 96.95 | 92.02 | 48.17 |
| DE20-1 | 21,198,048 | 20,327,412 | 6.36 | 6.1 | 95.89 | 0.03 | 97.18 | 92.53 | 47.5 |
| DE20-2 | 22,780,907 | 21,764,630 | 6.83 | 6.53 | 95.54 | 0.03 | 96.83 | 91.8 | 47.63 |
| DE20-3 | 21,326,283 | 20,389,833 | 6.4 | 6.12 | 95.61 | 0.03 | 97.13 | 92.41 | 46.81 |
| DP1-1 | 21,675,233 | 20,555,345 | 6.5 | 6.17 | 94.83 | 0.03 | 97.28 | 92.73 | 46.4 |
| DP1-2 | 23,741,848 | 22,663,092 | 7.12 | 6.8 | 95.46 | 0.03 | 97.48 | 93.22 | 47.35 |
| DP1-3 | 29,766,471 | 28,491,576 | 8.93 | 8.55 | 95.72 | 0.03 | 97.05 | 92.22 | 47.25 |
